# Toll-Like Receptor 4 Is Involved in Inflammatory and Joint Destructive Pathways in Collagen-Induced Arthritis in DBA1J Mice

**DOI:** 10.1371/journal.pone.0023539

**Published:** 2011-08-17

**Authors:** Matthias Pierer, Ulf Wagner, Manuela Rossol, Saleh Ibrahim

**Affiliations:** 1 Rheumatology Section, Medical Department II, University of Leipzig, Leipzig, Germany; 2 Department of Dermatology, University of Lübeck, Lübeck, Germany; University of Pittsburgh, United States of America

## Abstract

In rheumatoid arthritis, a significant proportion of cytokine and chemokine synthesis is attributed to innate immune mechanisms. TLR4 is a prominent innate receptor since several endogenous ligands known to activate the innate immune system bind to it and may thereby promote joint inflammation. We generated TLR4 deficient DBA1J mice by backcrossing the TLR4 mutation present in C3H/HeJ strain onto the DBA1J strain and investigated the course of collagen-induced arthritis in TLR4 deficient mice in comparison to wild type littermates. The incidence of collagen- induced arthritis was significantly lower in TLR4 deficient compared to wild type mice (59 percent vs. 100 percent). The severity of arthritis was reduced in the TLR4 deficient mice compared to wild type littermates (mean maximum score 2,54 vs. 6,25). Mice deficient for TLR4 were virtually protected from cartilage destruction, and infiltration of inflammatory cells was reduced compared to wt mice. In parallel to the decreased clinical severity, lower anti-CCP antibody concentrations and lower IL-17 concentrations were found in the TLR4 deficient mice. The study further supports the role of TLR4 in the propagation of joint inflammation and destruction. Moreover, since deficiency in TLR4 led to decreased IL-17 and anti-CCP antibody production, the results indicate a link between TLR4 stimulation and the adaptive autoimmune response. This mechanism might be relevant in human rheumatoid arthritis, possibly in response to activating endogenous ligands in the affected joints.

## Introduction

Rheumatoid arthritis is an autoimmune disease of diarthrodial joints with unknown etiology. T cells, B-cells and cytokines such as TNF alpha or IL-6 can be targeted therapeutically and are therefore important pathogenic components in the process of inflammatory mediated cartilage and bone destruction [1]. The primary factors responsible for an adaptive immune response targeting joints are not fully understood.

Toll-like receptors (TLR's), promote innate and adaptive immune responses, including induction of pro-inflammatory cytokines and matrix metalloproteinases [2].

Based on animal data, innate immune activation is indispensable for induction and chronicity of arthritis. As such, the skg arthritis or the IL-1ra −/− arthritis model are critical dependent on innate immune system stimulation. However, the clear nature of the ligands and the receptors for innate activation seem to widely differ between models. In skg mice, the development of arthritis is Dectin- dependent [3], while in the IL-1ra −/− model, disease is critically dependent on TLR-function, namely TLR4 [4] [5]. More evidence for a significant role of innate immune activation stems from streptococcal cell wall-induced arthritis, since MyD88 knockout mice are protected from joint inflammation in this model [6].

TLR's are highly expressed in synovial tissue from individuals with rheumatoid arthritis [7] and activation of synovial fibroblasts by TLR ligands may induce chemotactic attraction of immune cells [8]. Furthermore, an inhibitor of TLR4 has reduced symptoms in patients with moderate to severe rheumatoid arthritis in a preliminary phase 1 trial [9].

Beside TLR2, most endogenous ligands have been identified for TLR4, among them sHSP alphaA crystallin, HSPB8 [10] or Tenascin C [11]. Overall, the presence of these endogenous ligands in the synovial membrane supports a significant role for TLR4 in the pathogenesis of RA.

In collagen-induced arthritis, one of the most relevant animal models of RA, no data using genetically TLR4 deficient animals have been published yet. We therefore generated TLR4 deficient DBA1J mice and compared CIA development with wild type littermates.

## Materials and Methods

### Mice

Homozygous C3H/HeJ mice which carry a point mutation within the coding region of the Tlr4 gene resulting in a non-conservative substitution of a highly conserved proline by histidine at codon 712 [12] were backcrossed at least 8 times to DBA/1J mice (H-2^q^) to bring the TLR4^−/−^ mice to an arthritis-susceptible H-2^q^ background. The mice were intercrossed, and homozygous TLR4^−/−^ DBA/1 mice were obtained. Mice were bred and maintained at the animal facilities at the Medizinisch Experimentelles Zentrum, University of Leipzig, Germany and at the animal facility at the University of Rostock, Germany. The animals were fed rodent chow and water ad libitum.

#### Ethics Statement

The local ethics committee (Regierungspräsidium Leipzig, TVV31/05) approved all experiments.

### Genotyping

The C3H/HeJ TLR4 genotype was determined by PCR amplification and restriction length polymorphism analysis performed on isolated tail DNA. The TLR4 genotype was detected using the following primer pair (5′-CACgACgTTgTAAAACgACTgATgCATTTgTgATCTACTCg-3′) and (5′-ggCAgCAATggCTACATCA-3′) The gene amplification was performed in 25 µl of 2.5 mM MgCl_2_, 0.4 mM dNTP, 1 µM of each primer, and 1 U of AmpliTaq Gold DNA polymerase (Roche Applied Science, Mannheim, Germany) in PCR buffer for 33 cycles (94°C for 30 s; 56°C for 30 s; 72°C for 30 s). Genotype was identified by agarose gel electrophoresis after digestion with restriction enzyme PagI (Fermentas, St.Leon-Rot GERMANY) for 4 hours at 37° Celsius.

### Induction of CIA and assessment of arthritis

Mice were immunized with 50 µl of a 1∶1 (v/v) emulsion 0.1 M acetic acid containing 50 µg of chick type II collagen (CII; Chondrex) and 25 µl of incomplete Freund's adjuvant (DBA/1J (Chondrex, Redmond, WA, USA) at the base of the tail. Development of arthritis was assessed three times weekly by two blinded observers. The clinical severity of arthritis in each paw was quantified according to a graded scale from 0 to 4, as follows: 0, no swelling; 1, swelling in one digit or mild dorsal edema; 2, moderate swelling affecting several digits; 3, severe swelling affecting most digits; and 4, the most severe swelling and/or ankylosis. A total arthritis score per mouse was determined by summarizing the scores of all four extremities. The mean ± SEM values were determined. Incidence was defined as the cumulative percentage of arthritic mice out of the total number of immunized mice during the experiment.

### T cell proliferation assays and cytokine production

Spleens were removed under aseptic conditions and single-cell suspensions of mononuclear cells were prepared. The cells were washed three times in culture medium before being suspended to 2×10^6^ mononuclear cells per milliliter in round-bottom 96-well polystyrene microtiter plates (Nunc) in a total volume of 200 µl. The culture medium consisted of RPMI 1640 with Glutamax II (Invitrogen) supplemented with 50 IU/ml penicillin, 60 µg/ml streptomycin, and 5% inactivated FBS (all from Invitrogen). For lymphocyte stimulation, denatured pepsin-free T cell proliferation grade CII (Chondrex) was added to cultures at final concentration of 10 and 100 µg/ml. Cells were incubated at 37°C in humidified air with 5% CO_2_. After 3 days cells were pulsed with 10 µl of ^3^H methylthymidine (1 µCi/well; Amersham Pharmacia Biotech) and cultured for an additional 24 h. Cells were harvested onto glass-fiber filters (PerkinElmer) and [^3^H] thymidine incorporation was measured in a liquid beta scintillation counter. The results were expressed as cpm. For proliferation assays, cultures were done in triplicates. FACS analysis of T cell populations was conducted by surface staining for CD4 and CD25 (BD Biosciences) and for intracellular Foxp3 (eBioscience) according to published protocols. Culture fluid cytokine concentration of IL-17 was analyzed by cytometric bead array (CBA; BD Biosciences) according to the manufacturer's protocol.

### Measurement of collagen-specific Abs, anti CCP antibodies and cytokines

Sera were collected at day 34 by retro orbital blood drawing and at the day of sacrifice (day 91) from cardiac puncture blood drawings. After the samples had fully coagulated, they were centrifuged and the sera were stored at −80°C. Levels of CII-specific IgG2a and IgG1 were determined by ELISA using CII-coated (Chondrex) ELISA 96-well plates (Dynex Technologies) for capture and goat anti-mouse IgG2a and IgG1 Abs (Caltag) for detection. Absorbance (450 nm) was measured with an ELISA plate reader (Wallac). The measurements were done in triplicate. For each plate a standard consisting of pooled sera from arthritic mice was applied and used to determine relative antibody reactivity units.

Anti-CCP antibody titers were determined using the DIASTAT Anti-CCP2 kit (second generation peptides; Axis-Shield Diagnostics Ltd.) following manufacturer's instructions with the following modifications: for most experiments mouse sera were diluted 1∶10 or 1∶100 in sample diluent, and the secondary antibody was substituted with alkaline phosphatase-conjugated goat anti-mouse IgG (CALTAG Laboratories) diluted 1∶1000 in PBS. The kit standard was utilized without modification of the secondary antibody to maintain consistency between plates.

Cytokine concentrations for IL-6, IL-12p70, IL-12/23/p40, TNF-alpha, IL-10 and IL-17 were analyzed by cytometric bead array (CBA; BD Biosciences) according to the manufacturer's protocol.

### Histopathologic assessment

Mouse paws were fixed in 4% neutral buffered formalin (Sigma-Aldrich) and then decalcified, cut, and stained with H&E or with Nuclear Fast Rubine-Aniline Blue-Orange G. Four coronal sections 80 um apart were scored by two independent observers, at low power for cellular infiltration, exudation, and pannus, and at low (×10) and high (×100) power for bone erosion and cartilage destruction. A semi quantitative graded scale from 0 to 3 was used, as follows: 0, no changes; 1, mild changes; 2, moderate changes; and 3, most severe changes observed in the experiments. Cartilage destruction was determined as loss of cartilage in relation to the total cartilage area. A mean score for each animal was determined for each parameter, and the scores were averaged to determine group means.

### Statistics

We used Prism 5.0 to calculate means, SEM and statistical tests. Differences between groups were calculated using two-tailed t test for antibody responses, T cell proliferation measures and cytokine concentrations. The Mann-Whitney U test was used for differences between histological scores. Differences in arthritis incidence were analyzed using Fishers exact test. P values less than 0.05 were considered significant.

## Results

### CIA is alleviated in TLR4 deficient mice

To investigate the role of TLR4 in murine collagen-induced arthritis, TLR4 deficient C3H/HeJ mice were backcrossed for at least 7–10 generations onto the CIA susceptible DBA/1J background. DBA1/J-C3H/HeJ/C3H/HeJ mice proved to be lps hypo-responsive since splenocytes did not produce TNF alpha in response to lps while splenocytes derived from TLR4 wt/wt littermates produced normal amounts (data not shown).

**Figure 1:**
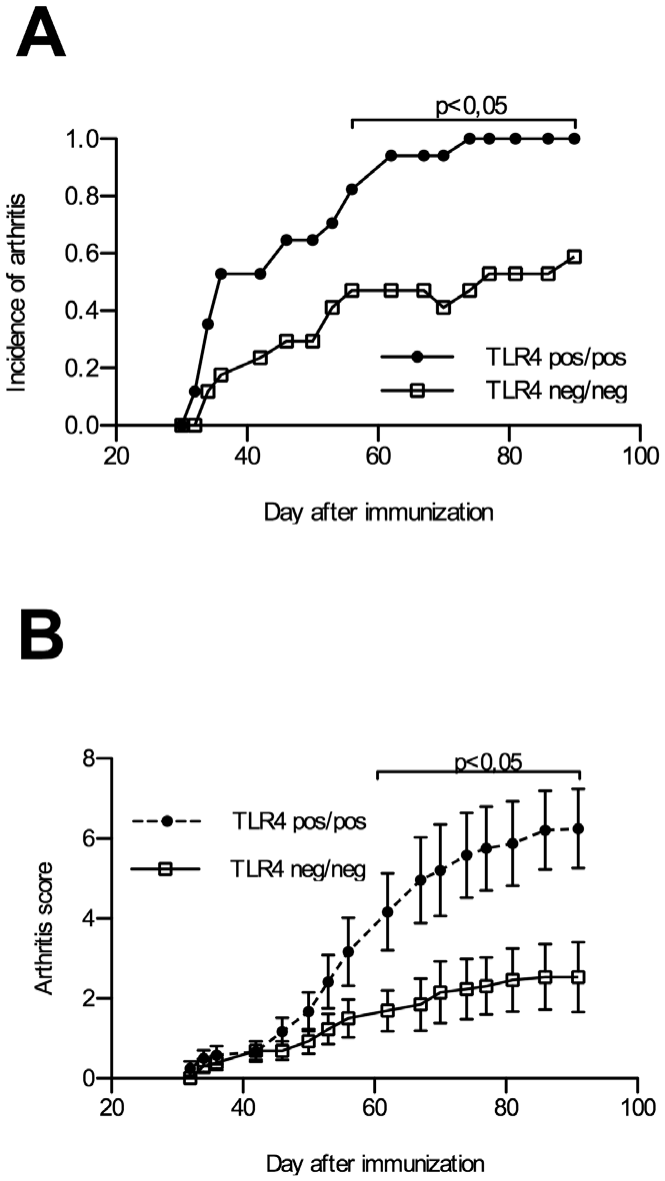
Incidence and severity of arthritis. Suppressed incidence (A) and severity (B) of collagen induced arthritis (incomplete Freund's adjuvant) in TLR4 defective DBA/1J (n = 17) compared to TLR4 pos/pos (n = 17) DBA1/J mice.

For collagen-induced arthritis in this strain, a protocol with incomplete Freund's adjuvant without heat-killed mycobacteria was used in order to avoid potential TLR4 stimulation in wildtype mice by mycobacterial substances [13]. DBA1/J mice deficient for TLR4 and littermates carrying the wild type TLR4 alleles were immunized with collagen II and were followed over a time period of 90 days. As shown in Figure 1A, in the absence of functional TLR4 the clinical severity of arthritis was lowered. Overt arthritis developed at about the same time but showed a significantly reduced incidence reaching only 67 percent over the observation time compared to a 100 percent incidence in the wt groups. Arthritis severity, as defined by the scoring method described in the M&M section, was significantly milder in TLR4 defective mice with a mean maximal score of 2,54 compared to 6,25 for the wt groups (Figure 1B). This suppressive effect of TLR4 deficiency became obvious approximately 2 weeks after arthritis onset and was sustained in the chronic phase up to the end of the observational period of 90 days.

### TLR4 deficient mice are protected against joint destruction

**Figure 2:**
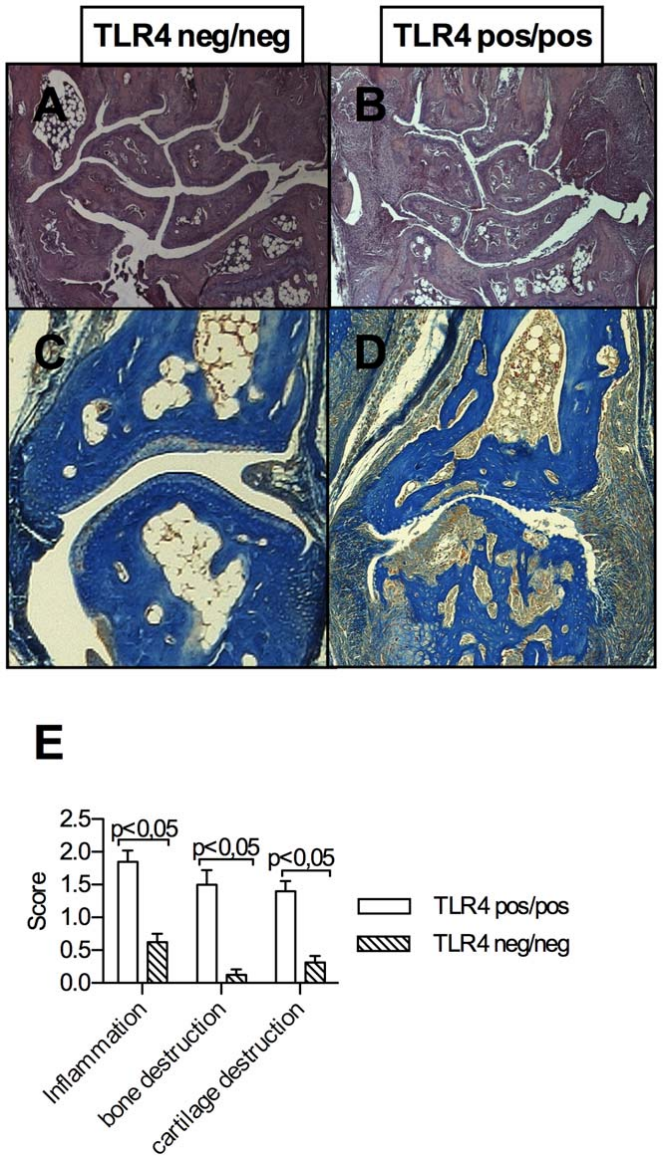
Histological joint destruction in paw sections. Synovial inflammation is suppressed in CIA in DBA1J TLR4 negative mice (A, C) compared to wt mice (B, D). Representative front paw sections showing wrist and carpal joints and stained in H&E, original magnification ×10 are shown in (A) and (B) and interphalangeal joints with extensive pannus formation and bone destruction stained with Nuclear Fast Rubine-Aniline Blue-Orange G in (C) and (D), original magnification ×100. Histological joint scores reflecting significantly suppressed inflammation and cartilage and bone destruction in TLR4 defective mice are shown in (E).

The clinically observed amelioration of arthritis was reflected by suppression of joint destruction in the histological assessment of H&E stained paw sections (Figure 2). Staining with Nuclear Fast Rubine-Aniline Blue-Orange G shows significantly less bone erosion and infiltration of pannus tissue in TLR4 deficient mice. Mice deficient for TLR4 were virtually protected from cartilage destruction in IFA induced CIA and infiltration by inflammatory cells was significantly reduced in TLR4 defective mice compared to wt mice. The overall inhibition of synovitis in TLR4 deficient animals is shown in Figure 2E.

### Autoantibody production in TLR4 deficient arthritic mice

**Figure 3:**
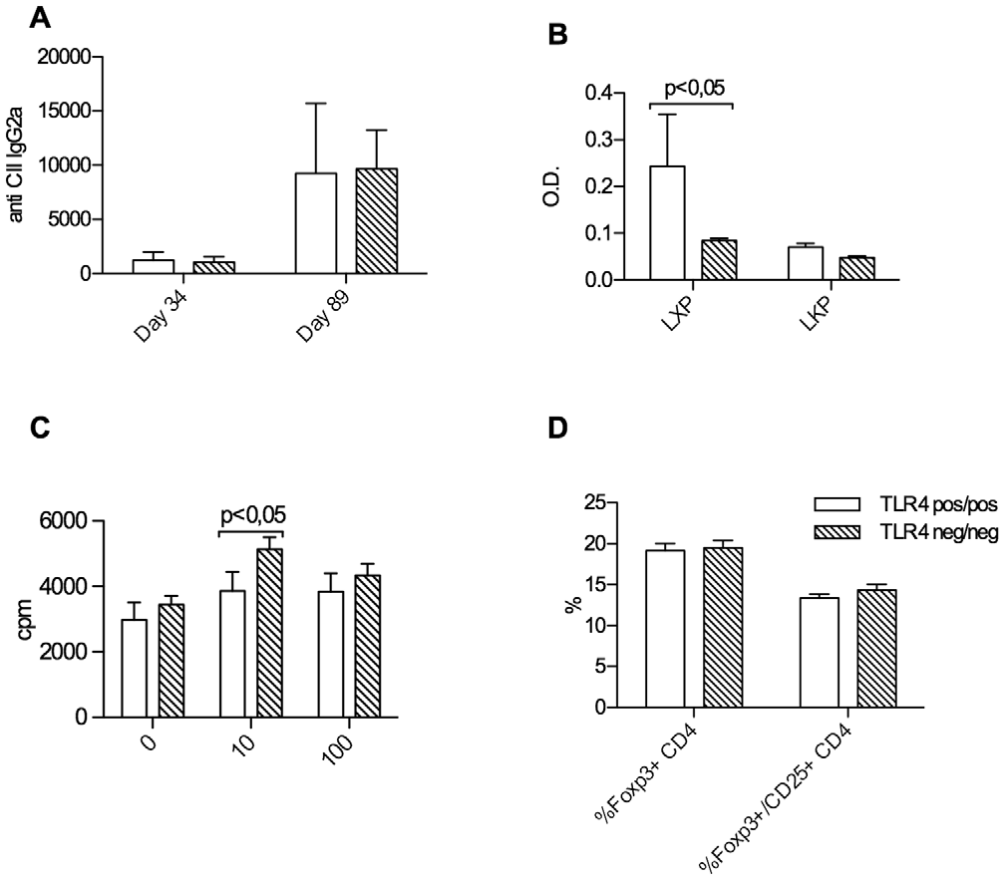
B and T cell responses. No significant difference in peripheral blood anti-collagen type II antibody concentrations of the IgG2a subclass at days 34 and 89 in TLR4 negative versus positive groups of mice (A). Anti-cyclic citrullinted peptide (CCP) antibody concentrations (anti-LXP) are significantly higher in TLR4 positive groups of mice at day 34 after immunization, while anti-non-citrullinated controle peptide antibody concentrations are comparable (B). T cell recall responses in TLR4 negative mice were significantly stronger than in wt mice at the lower tested antigen concentration (C). No influence on foxp3 positive Treg numbers was seen (D).

The humoral response against collagen II is crucial in the development of erosive arthritis. Despite the observed clinical differences, however, the anti-collagen type II antibody concentrations of the Th1 dependent IgG2a subclass determined in the peripheral blood at days 34 and 89 did not differ between TLR4 negative and positive mice (Figure 3A). For anti-collagen type II IgG1 subclass antibodies, again no differences were measured (data not shown).

The second pathogenetically relevant humoral response in murine collagen induced arthritis is targeted against citrullinated side chains of endogenous proteins [14]. In accordance with the clinical picture of an attenuated joint destruction in TLR4 deficient mice, anti-citrullinated peptide antibody (ACPA) concentrations (anti-LXP) were significantly lower in the knockout mice compared to wild type littermates at day 34 after immunization, while anti-non-citrullinated control peptide antibody concentrations were comparable (Figure 3B).

### T cell response and cytokine production in TLR4 deficient arthritic mice

The response of CD4 positive T cells against peptide antigens derived from the injected type II collagen is central for the development of murine arthritis. When this response was tested ex vivo by measurement of 3H thymidine incorporation following re-stimulation with collagen II, a higher rate of proliferation was unexpectedly detected in the TLR4 deficient group (Figure 3C), which was reflected also by higher Interferon gamma concentrations in vitro (data not shown). In view of the attenuated severity of arthritis in those mice, increased proliferation of suppressive or regulatory T cells was a possible explanation for this result. When those cells were quantified, however, no difference between TLR4 deficient mice and wild type littermates in circulating Treg numbers (as defined by CD25 positivity and intracellular Foxp3 expression) was seen (Figure 3D).

**Figure 4:**
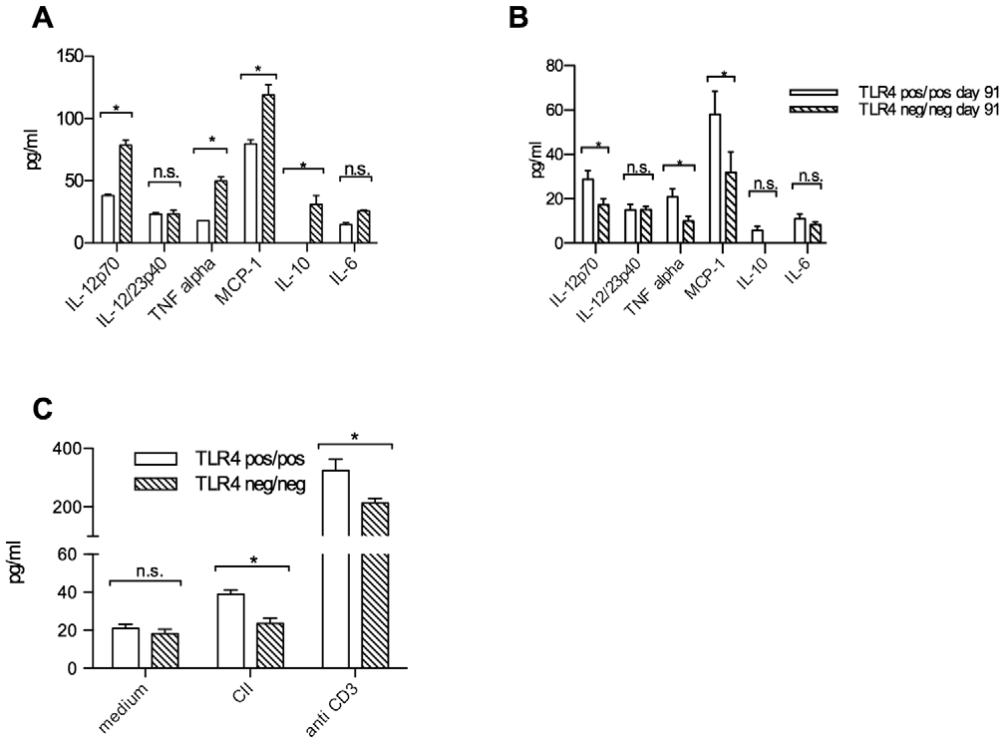
Cytokines in sera and in vitro splenocyte cultures. Serum cytokine concentrations measured at days 34 (A) and 91 (B) in TLR4 negative and TLR4 positive groups of mice. Significantly higher IL-12p70, TNF alpha, MCP-1 and IL-10 concentrations in TLR4 negative groups of mice at day 34 after immunization, while at day 91 IL-12p40, TNF alpha and MCP-1 were higher in TLR4 positive mice. (C) Interleukin17 concentrations are reduced in TLR4 deficient in vitro spleen cell cultures after 48 hours in the presence of type II collagen (100 ug/ml) or anti-CD3 antibodies (1 ug/ml)(*p<0,05).

In an attempt to characterize the involved immune pathways, serum concentrations of a panel of cytokines were determined. Again somewhat unexpectedly, concentrations of the pro- inflammatory cytokines IL-12p70, TNF alpha, MCP-1 and of the regulatory cytokine IL-10 were higher in TLR4 negative mice at day 34 after immunization (Figure 4 A). However, at day 90 after immunization, in the chronic stage of arthritis, the pro-inflammatory cytokines IL-12p70, TNF alpha and MCP-1 were suppressed in TLR4 deficient mice compared to wt mice (Figure 4 B).

In contrast, Il-17 concentrations, measured in vitro in spleen cell cultures stimulated with either type II collagen or anti-CD3 antibodies, were higher in cultures derived from TLR4 positive mice (Figure 4 C), suggesting that increased numbers of differentiated Th17 cells were present in those ex vivo analyses, and presumably also in vivo.

## Discussion

Recent evidence supports the pathogenic role of innate stimulation via Toll-like receptors in animal models of arthritis like SCW Arthritis, collagen type II antibody induced arthritis or KB/N serum transfer arthritis. For one of the most relevant models of rheumatoid arthritis, the collagen induced arthritis (CIA) model, no data using genetically deficient animals have been published.

Here we show that TLR4 deficient DBA mice develop collagen induced arthritis with lower incidence and decreased severity, thereby supporting a role for TLR4 in autoimmune arthritis.

In parallel to the reduced clinical severity in TLR4 deficient mice, their anti-CCP antibody concentrations are lower. In humans, it has recently been shown, that an increased frequency of Th17 cells is associated with anti-CCP positive disease [15]. Consequently, the decreased IL-17 production of TLR4 deficient splenocytes upon re-challenge with collagen II could be associated with the observed decrease in anti-CCP antibodies, thereby indicating a possible involvement of TLR4 in these responses. In line with this result, the control of B cell responses has been shown to depend on TLR stimulation [2], however, others have shown that TLR derived signals are dispensable [16]. The chemical nature of the antigens has been shown to contribute to this discrepancy [17] and such differences might play a role for the observed different effect of TLR4 deficiency for the generation of anti- citrullinated peptide antibodies in contrast to identical generation of anti- collagen antibodies.

In contrast to the reduced clinical severity of arthritis, however, a more vigorous immune response to immunization with collagen II seems to occur in TLR4 deficient mice, since both the serum concentrations of the proinflammatory cytokines IL-12p70, TNF alpha and MCP-1 (day 34) and the T cell responses against collagen II are higher in this group. The augmented T cell response despite the attenuated reduced clinical severity cannot be attributed to Treg activation, since no numerical expansion of suppressive Tregs was observed.

An alternative hypothesis is that the immune response against collagen II is dissociated from the progression of joint disease, while a Th17 dependent response against citrullinated peptide is a driving force of destruction. This would also imply, that TLR4 deficiency is attenuating this anti-citrulline response, and therefore suggest an involvement of TLR4 signalling in this response. Endogenous ligands might play a relevant role in this process. In line with this, direct TLR4 stimulation in CD4 T lymphocytes has been shown to promote T helper 17 responses and to aggravate the evolution of autoimmune diseases [18], indicating that the observed decrease in IL-17 production may indeed contribute to the suppressed arthritis in TLR4 defective mice. Results showing that TLR4 together with Dectin-1 is necessary for he induction of Th17 cells by mycobacteria have suggested a similar mechanism [19]. However, reduced anti-CCP antibody response might be a consequence of otherwise suppressed arthritis in TLR4 deficient mice.

Taken together, the results thus suggest that after immunization with collagen II, with the exception of ACPA generation and IL-17 production, most early immune activation events occur in a TLR4- independent manner, including anti- collagen antibody formation and production of pro-inflammatory mediators. However, the strong immune reaction in TLR4 deficient mice does not induce joint inflammation of the same magnitude as in TLR4 wt mice. This data further support a role for endogenous ligands for TLR4 and may link TLR4 to ACPA generation and IL-17 production.

The findings are in line with results of TLR4 inhibition with a naturally occurring antagonist in the collagen induced arthritis model and the IL-1ra −/− model [4], the KRN serum transfer arthritis [20] as well as with results from the antigen-induced arthritis model [11], for which tenascin was identified as critical endogenous ligand of TLR4.

The results further strengthen the role of TLR4 in the propagation of intra-articular inflammation and joint destruction. Decreased IL-17 and ACPA production due to missing innate response to endogenous TLR4 ligands may be reason for the reduced severity of CIA in TLR4 deficient mice.
